# Airway sensory systems: breathing new life into microbiota-brain communication

**DOI:** 10.3389/fncel.2025.1632805

**Published:** 2025-11-25

**Authors:** Ritu Mann-Nüttel, Marie Armbruster, Shivani Mandal, Paul Forsythe

**Affiliations:** Alberta Respiratory Centre, Pulmonary Division, Department of Medicine, University of Alberta, Edmonton, AB, Canada

**Keywords:** asthma, COPD, lung-brain axis, mental health, olfaction

## Abstract

The essential role of the lungs in gas exchange necessitates exposure to possible threats from a dynamic external environment. To protect life-critical functions the airways contain multiple systems that monitor the inhaled environment and elicit appropriate defensive responses. As such the airways represent a key sensory surface with multiple signaling pathways to the brain. Despite the presence of rich and diverse bacterial communities in both upper and lower airways, the respiratory tract has been relatively overlooked compared to the gut regarding its potential as an interface between microbes and the central nervous system. This review draws attention to the respiratory system, specifically the nasal cavity and lungs, and the evidence supporting a microbiota-airway-brain axis. We highlight the olfactory system and the role of the lungs as a sensory organ, monitoring the inhaled environment, as clear examples of airway-brain communication and identify how these communication pathways can be engaged by microbes. We also outline the relationship between the airways and mental health and present the case that the nasal and lung microbiota should be considered alongside that of the gut as potential influencers of brain function, mood, and behavior.

## 1 Introduction

The concept of a microbiota-gut-brain axis has been seen as a breakthrough in our understanding of mental health and the pathogenesis of neurological disorders (Forsythe et al., 2010; Forsythe et al., 2016; Lachmansingh et al., 2024). Yet the role of the brain in maintaining optimal fitness requires monitoring (afferent signaling) and subsequent adjustment (efferent signaling) of all physiological systems. Furthermore, all surface barriers (skin and mucosa of the gastrointestinal, reproductive, and respiratory tracts) have a population of commensal microbes, and thus there are multiple potential microbiota-brain axes within the brain-body network. Here we wish to draw attention to the respiratory system, in particular the lungs and nasal cavity, and the evidence for a microbiota-airway-brain axis.

**FIGURE 1:**
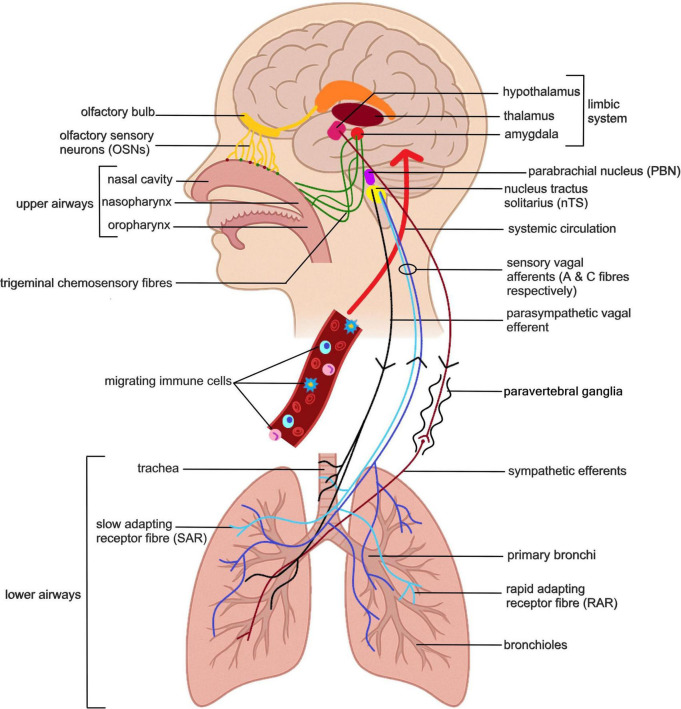
Airway-brain communication pathways. Major sensory signaling from the airways to the brain occurs via olfactory neurons and the trigeminal nerve in the nasal cavity, and vagal sensory neurons in the lower airways. The vagal and trigeminal sensory neurons enter the brain via the NTS and trigeminal nucleus, respectively, and via the parabrachial nucleus can influence wider brain circuity including regions such as the amygdala and hypothalamus. In addition to neural signals, immune changes in the airway can be signaled to the brain via circulating lymphocytes or cytokines via circumventricular organs, a permeable blood brain barrier, or active uptake.

A relationship between airways and brain is clear. At the most prosaic level the lungs are vital for gas exchange between the environment, bloodstream, and the brain- the most oxygen-hungry organ in the body. However, concomitant to the role in gas exchange, the lungs must monitor the inhaled environment for O_2_/CO_2_ levels, as well as potential threats from pathogens and contaminants such as volatile chemicals and particulates (Garg et al., 2019). This critical sensory function involves constant bi-directional communication with the brain (Figure 1). Similarly, the nasal cavity, beyond its role in humidifying and warming air, contains the chemosensory olfactory system. This system communicates critical environmental information to the brain, impacting behaviors like threat awareness and eating habits (Canteras et al., 2008; Bienenstock et al., 2018; Kavaliers et al., 2022; Li and Wilson, 2024).

In addition to well established behavior regulating communication between the airways and brain, we now know that both the upper and lower airways are exposed to rich and diverse bacterial communities. Yet, perhaps because of a much lower microbial load compared to the gut, the respiratory tract has been relatively overlooked regarding microbial modulation of brain function and behavior.

In this review we focus on two key sensory organs in the airways—the nasal cavity and lungs. We outline the communication pathways linking the respiratory system and the brain, identify the relationship between the airways and mental health, and present the case that the nasal and lung microbiota should be considered alongside that of the gut as potential influencers of brain function, mood, and behavior. By integrating both nasal and pulmonary microbiota within the broader framework of airway–brain communication, we aim to provide a comprehensive perspective of this emerging field.

## 2 Airway microbiota

### 2.1 Microbial niches of the airways

The airways serve as an interface between external and internal environments and host distinct microbial communities that vary across regions, offering an array of potential pathways for communication with the brain. The airways can be divided broadly into two regions: the upper airway and the lower airway. The upper airway consists of the nasal cavity, sinuses, nasopharynx, oropharynx, and laryngopharynx, while the lower airway encompasses the trachea and the lungs (Sahin-Yilmaz and Naclerio, 2011). The differences of pH, temperature, oxygen concentration and mucus secretion along the airways lead to the development of distinct and specialized microbiota niches (Schenck et al., 2016).

The nasal cavity is the first point of contact between the external environment and the respiratory tract. With a continuous inflow of air and dust particles, it is also the airway region with the greatest exposure to microorganisms. Therefore, it is not surprising that the nasal cavity is colonized by a relatively diverse range of microbes including bacteria, archaea, fungi and viruses (Schenck et al., 2016). Bacteria are the major constituent of the nasal microbiome with the human nasal cavity containing an estimated 10^4^ bacterial strains and 10^6^–10^8^ bacteria per gram of tissue (Bomar et al., 2018). The resident nasal microbiota in healthy adults is largely distinct from that found in the oral cavity, while sharing some similarities with that of the skin microbiota, with most abundant phyla of the nasal microbiota being *Actinobacteria*, *Firmicutes* (*Bacillota*), *Proteobacteria* and *Bacteroidetes* (*Bacteroidota*) (Koskinen et al., 2018).

General acknowledgment that the healthy lung has a resident microbiome is a relatively recent development. Previously, the lower airways were considered sterile, and it is only through use of culture-independent techniques that the rich and diverse, yet relatively sparse, bacterial community of the lungs has been revealed. *Bacteroidetes* and *Firmicutes* are the most abundant phyla detected in the lower airways with *Prevotella*, *Veillonella*, and *Streptococcus* the prominent genera. In terms of biomass the lung microbiota [10^3^–10^5^ bacteria per gram of tissue (Mathieu et al., 2018)] is markedly lower than that of the nasal cavity (Bomar et al., 2018).

The composition of the lung microbiota most closely resembles that of the mouth when compared to other body sites (Bassis et al., 2015). Notably, the nasal microbiota makes limited contribution to lung colonization in healthy individuals (Bassis et al., 2015; Yagi et al., 2021). However, constituents of the nasal microbiota such as *S. aureus* and *M. catarrhalis* are respiratory pathogens in some inflammatory lung diseases including pneumonia. Based on the similarity between mouth and lung microbiomes, it has been hypothesized that the lung is colonized following aspiration of oropharyngeal secretions, micro-aspiration, or direct dispersal along the airway mucosa (Proctor and Relman, 2017). However, a comparison of the lung and oropharyngeal microbiota (Morris et al., 2013) identified a significantly higher abundance of specific bacteria in the lungs than would be expected if they originated from the oropharynx. This suggests that the oropharynx is not the only contributor to the lung microbiota and/or the microenvironment of the lungs drives site-specific reproduction and selective growth of certain organisms (Bassis et al., 2015; Yagi et al., 2021; Morris et al., 2013). It is very likely that there are strong selective pressures on growth of bacteria in the lower airways as they present a relatively inhospitable environment to bacteria. Unlike the microbe-favoring conditions of the gut, oral cavity, and upper airways, the lower airways have limited nutrient availability and high oxygen stress. While epithelial surfaces of the trachea and bronchi are mucus-covered, like the GI tract, the alveoli which make up most of the lung surface area, are coated with surfactant, a lipid-protein complex with bacteriostatic activity (Chaby et al., 2005). Furthermore, the lungs have a resident macrophage population that act as sentinels at luminal surfaces of the alveoli, tasked with phagocytosing and killing bacteria (Aegerter et al., 2022).

### 2.2 Factors influencing the airway microbiota

The airway microbiota composition of an individual will be determined by several intrinsic and extrinsic factors. Newborns inherit much of their microbiota from their mothers, with delivery mode shaping the initial microbial community across body sites (Dominguez-Bello et al., 2010). Infants delivered via vaginal birth are first exposed to vaginal microbes while initial exposure of those delivered via cesarean-section is to the maternal skin microbiota (Dominguez-Bello et al., 2010). The microbiota of the upper airways, like the intestine, is highly dynamic over the first 2–3 years of life and influenced by exposures such as breast-feeding, attendance at day care, and antibiotic treatment (Bosch et al., 2017). However, the equivalent trajectory of the lower airway microbiota has yet to be delineated.

Throughout life, the airway microbiota composition will be influenced by microbes from the air (aerobiome) and additional components of the inhaled environment. Exposure to air pollutants has been shown to alter microbiota composition (Mariani et al., 2018). Smoking cigarettes also modulates the airway microbiome with smokers having a more diverse oro- and nasopharyngeal microbiota compared to non-smokers (Charlson et al., 2010). Even passive cigarette smoke exposure can alter the airway microbiome as evidenced by increased levels of bacterial pathogens in children with smoking parents (Brook and Gober, 2008). Seasonal fluctuation in temperature and humidity can also impact the airway microbiota by favoring the growth of some bacteria over others (Hassoun et al., 2015).

Intrinsic host factors will also determine relative reproduction rates of microbes throughout the airways. Lung-resident innate immune cells, such as macrophages, dendritic cells, mast cells, natural killer and innate lymphoid cells, provide the first line of antimicrobial immunity (Seo et al., 2020; Zazara et al., 2022), with adaptive immune cells including type 1 T helper (Th1), Th2, Th17 and regulatory T cells (Treg) also contributing to host defenses (Vats et al., 2024). Consequently, genes involved in mucosal immunity including pattern recognition receptors, cytokine/chemokine signaling and barrier defense may significantly influence the airway microbiota. For example, genome wide association studies (GWAS) showed a positive association between mucosal immunity genes and the relative abundance of upper airway microbiome (Igartua et al., 2017) while polymorphisms in microbe associated molecular pattern (MAMP) receptors, CD14 and Toll-like receptor 2, influence the microbial composition in subjects with asthma (Losol et al., 2021).

### 2.3 The airway microbiota in respiratory disease

Changes in the microbial ecology of the airways have been observed in association with diseases. Alterations in the respiratory microbiota have been linked with local inflammation, tissue damage and lung diseases including cystic fibrosis (Lynch and Bruce, 2013), cancer (Goto, 2022), idiopathic pulmonary fibrosis (Amati et al., 2022), chronic obstructive pulmonary syndrome (Budden et al., 2019), and asthma. A relationship has been identified between altered nasal microbiota and both onset and severity of asthma. Increased colonization of the nasopharynx by *Streptococcus* spp. in the first year of life was highly associated with subsequent development of asthma (Tang et al., 2021) while children with asthma have a nasopharyngeal microbiota distinct to that of non-asthmatics (van Beveren et al., 2024). Bronchial brushing and bronchial alveolar lavage have demonstrated that lungs microbiota also differs between healthy individuals and those with chronic inflammatory lung disease (Hilty et al., 2010). COPD and asthmatic adult patients display a significant increase of *Proteobacteria*, particularly *Haemophilus* spp., in both the upper and lower airways compared to controls and has been correlated with asthma severity in both induced sputum and nasopharyngeal samples (Zhang W. et al., 2024). *Proteobacteria* have also been identified as increased in children with asthma. The genus *Veillonella* has also been associated with increased risk of asthma exacerbation and immune perturbation in infants (Tang et al., 2021) and associated with asthma severity in adults (Zhang W. et al., 2024). Conversely, *Corynebacterium* and *Staphylococcus* have been associated with lower levels of asthma-related inflammation, suggesting a protective role (van Beveren et al., 2024). However, while there have been consistent observations of altered nasal and lung microbiota in asthmatic and COPD patients, it is still not clear if such changes are a cause or a consequence of the disease.

Above we established that the airways have a rich and diverse microbiota, the composition of which is determined by extrinsic and intrinsic factors and is altered by disease states. As such, microbes represent a dynamic component of the airway lumen environment. Next, we discuss the systems that monitor that environment and play key roles in maintaining homeostasis including signaling to the brain to direct necessary adaptive or protective responses.

## 3 The sensory airways

### 3.1 Nasal sensory systems

**FIGURE 2:**
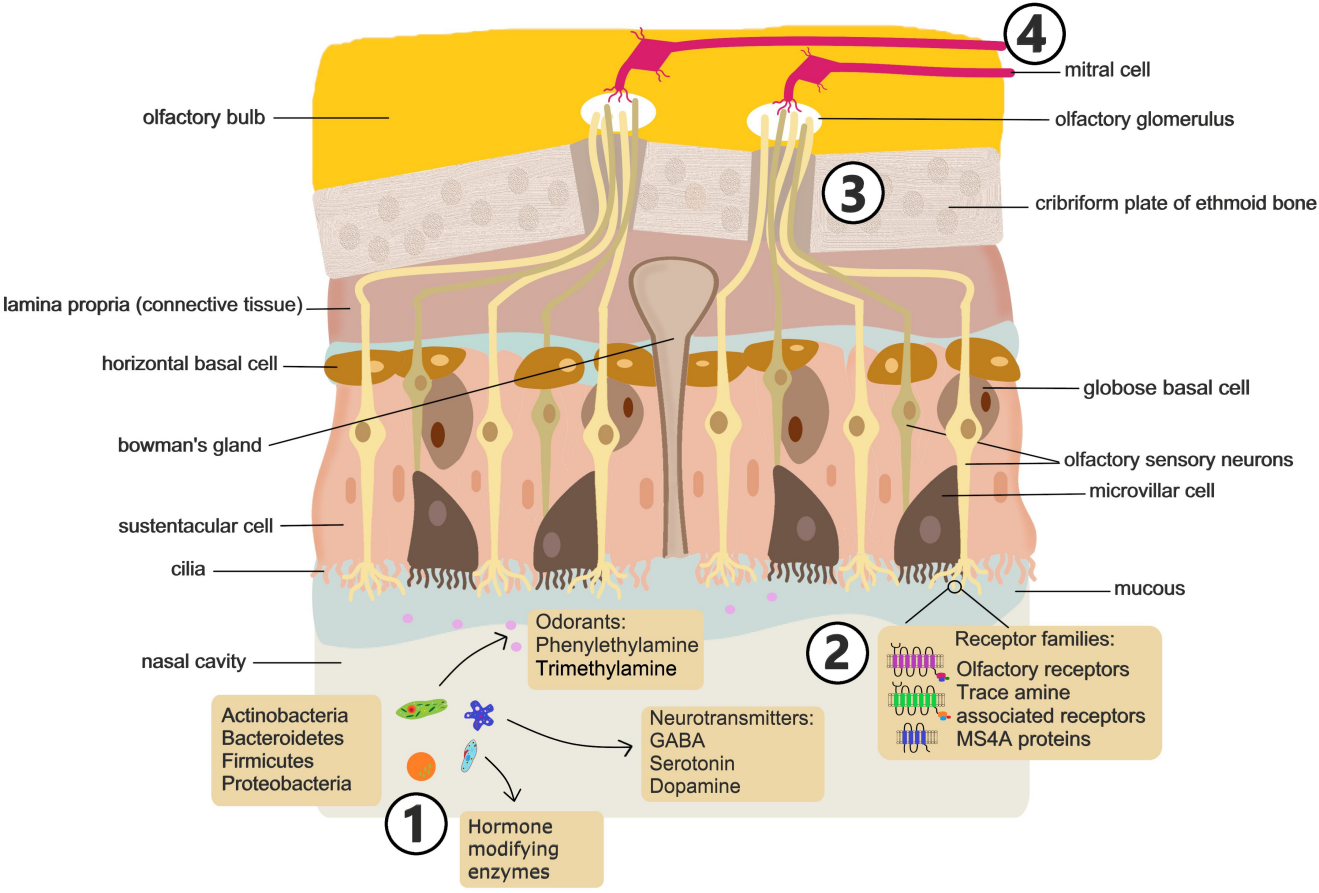
Sensing of microbial signals in the nasal cavity. Future studies should address: (1) how nasal microbiome composition is altered in mood disorders and how disruption of the composition or exposure to specific microbes influences behavior. (2) What metabolites, volatile compounds and metabolic enzymes are associated with mood altering microbiomes and what specific ligand receptor interactions drive microbial activation of olfactory neurons. (3) Which subsets of olfactory neurons drive behavioral effects of microbes. (4) Which brain circuits are modulated by microbial stimulation of olfactory neurons and the consequences for brain function and behavior.

The nasal cavity is the conduit to the airways, acting to humidify and warm inhaled air as well as protect the respiratory tract through mucociliary clearance. The nasal cavity also contains the olfactory system, a sensitive chemosensory network (Figure 2) that communicates critical information about the environment and influences a range of behaviors, eating habits, and threat awareness (Sharma et al., 2019; Ullah et al., 2024). These processes underscore the role of the olfactory system as a critical pathway for environmental signals to influence brain activity and behavior.

Olfactory function is facilitated by 6–10 million sensory neurons in the olfactory epithelium located in the dorsal and posterior section of the nasal cavity extending along the nasal septum and turbinates (Sharma et al., 2019). The olfactory epithelium is pseudostratified and consists mainly of horizontal and globose basal cells, sustentacular cells, and the olfactory sensory neurons (OSN). Mature OSN are bipolar cells with multiciliated dendrites extending into the nasal cavity (Ullah et al., 2024). The cilia on the apical surface of OSN express olfactory receptors (OR). OR are coded for by the largest multi-gene family found in mammals, consisting of around 400 genes in humans and over 1,000 in mice (Godfrey et al., 2004; Malnic et al., 2004). These G protein-coupled receptors bind several different molecular ligands with high-affinity. Odorant molecules bind to olfactory receptors on sensory neurons, initiating electrical signals that travel to the olfactory bulb and thence to higher-order brain regions, influencing threat perception and behavior. Generally, only one OR type is expressed on a single OSN. Distinct odors are perceived by the brain in the form of a code generated by the activation of multiple OR on different neurons (Kaur et al., 2014).

Beyond the classical OR, olfaction is also mediated by a distinct chemo-signaling system that is dependent on Trace Amine Associated Receptors (TAARs) (Liberles and Buck, 2006). TAARs are present on OSN of rodents and human, as well as on neurons and glia of the brain. While distinct from classical OR, TAARs are capable of sensing volatile odorants and a number of amines, such as cadaverine, that humans perceive as foul or aversive (Liberles, 2015). TAARs are also involved in the recognition of some classical amine neurotransmitters such as dopamine, serotonin (5-HT) and histamine (Liberles, 2015).

There is also evidence that another, non-canonical, non-GPCR, chemosensory receptor family plays a role in mammalian olfaction (Greer et al., 2016). The MS4A family of proteins are expressed in many cell types and tissues throughout the body, including peripheral immune cells and microglia (Villegas-Llerena et al., 2016; Mattiola et al., 2021) and a number of human diseases including Alzheimer’s disease and asthma have been strongly linked to polymorphisms in MS4A genes (Arthur et al., 2020; Lutz et al., 2020). In mice, these chemosensory receptors are also expressed within a specialized subsystem of olfactory sensory neurons and, unlike classical OR, more than one MS4A family member is expressed per neuron. The MS4A family detects and responds to behaviorally relevant odorants like pheromones and fatty acids (Greer et al., 2016). However, the contribution of this chemosensory receptor family to human olfaction remains to be determined.

More general sensory information from the nasal cavity is transmitted via the trigeminal nerve. Also known as the 5th cranial nerve, the trigeminal nerve has a branch (Maxillary) that innervates the nasal cavity with fibers projecting to the lateral parabrachial complex where signals are then transmitted to other brain regions including the amygdala (Aucoin et al., 2023; Shusterman, 2023). In addition to providing general sensory information regarding touch, pain, and temperature, the trigeminal nerve is responsible for awareness of airflow through the nasal cavity (Oleszkiewicz et al., 2018) and plays a role locating the source of odors (Kobal et al., 1989).

### 3.2 Lung sensory nerves

**FIGURE 3:**
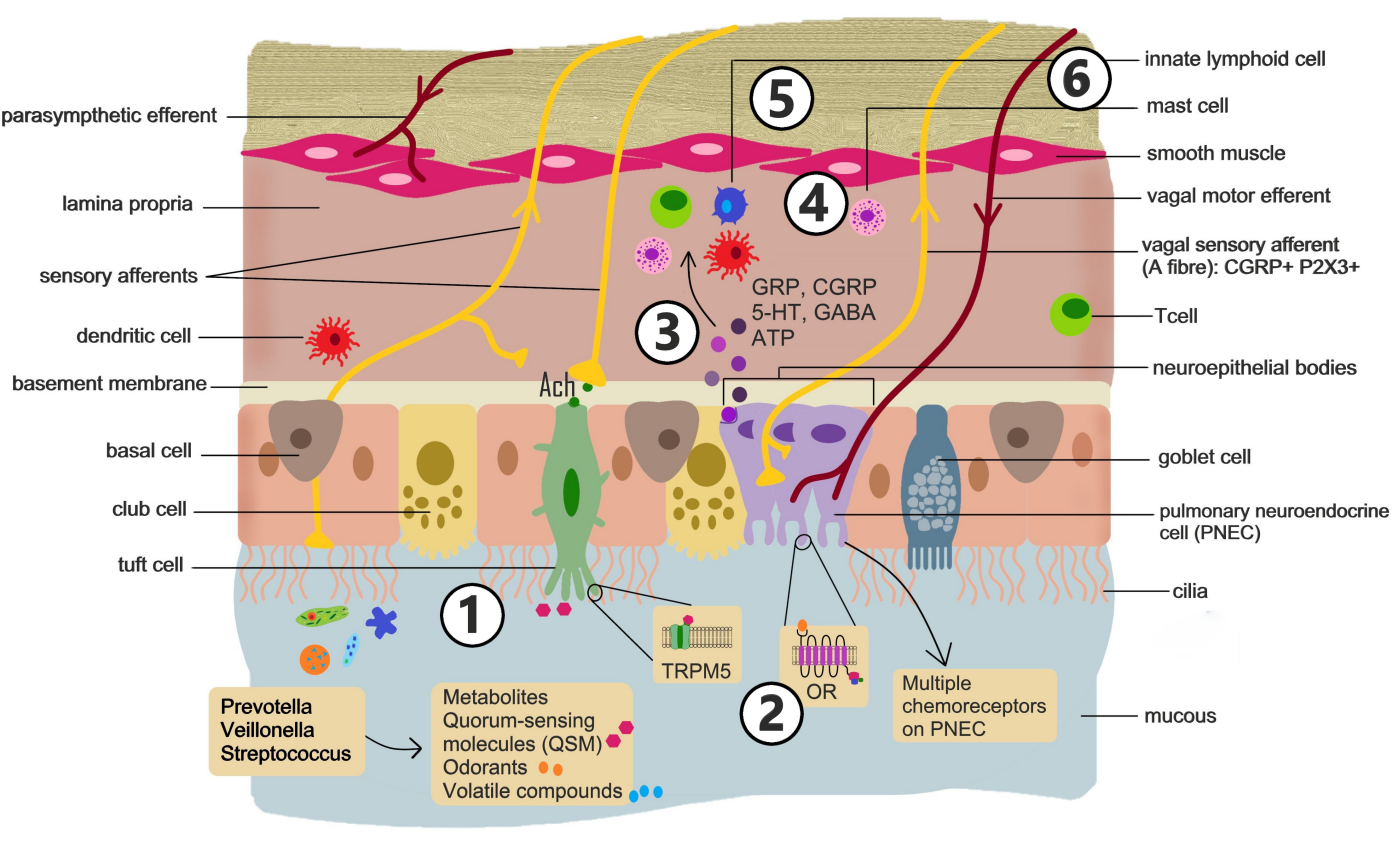
Sensing microbial signals in the lungs. Confirmation of a microbiota-lung-brain axis will require understanding of: (1) How the lung microbiome composition is altered in mood disorders and how disruption of the composition or exposure to specific microbes influences behavior, and how stress exposure influence the lung microbiota profile. (2) Specific chemosensory cells and receptors in the lung epithelium involved in detecting microbe derived signals. (3) How chemosensory cells in the lung communicate and modulate function of neurons and immune cells. (4) How immune cells and cytokines they produce communicate influence neuroinflammation. (5) Specific neuronal subtypes involved in responding to chemosensory cues from microbes. (6) The pathways though which these neurons influence brain circuitry involved in emotional responses and mood.

The lungs can detect diverse aerosol inputs (e.g., pathogens, allergens and pollutants) and translate them into appropriate physiological responses, such as changes in breathing patterns, cough, bronchoconstriction, and mucous production, that maintain mucosal defense and the critical role of the lung in gas exchange (Garg et al., 2019). These sensory responses depend on the coordinated interactions of the nervous, immune and endocrine systems (Figure 3).

The lungs are highly innervated, containing sympathetic, parasympathetic and sensory fibers (Su et al., 2022; Taylor-Clark and Undem, 2022). Sympathetic innervation of the lungs is supplied by ganglia stemming from the upper thoracic segments of the spinal cord, providing noradrenergic regulation of bronchial submucosal glands and blood vessels. The vagus (10th cranial) nerve provides all parasympathetic, and most sensory nerve fibers, to the airways. The internal branch of the superior laryngeal nerve provides the major sensory innervation of the larynx and caudal pharynx while the recurrent laryngeal nerve provides the majority of afferents to the trachea and lung.

The vagal afferent neurons that innervate the entire respiratory tract project to the brainstem, synapsing with second order neurons in the nucleus tractus solitarius (NTS) and paratrigeminal complex (Taylor-Clark and Undem, 2022). The NTS and paratrigeminal complex have projections to the parabrachial nucleus (PBN) that in turn signals regions, such as the amygdala and hypothalamus (Huang et al., 2025). The NTS also projects to the thalamus specifically the ventromedial nucleus and midline thalamic nuclei. Acting via the thalamus signals from the NTS can also influence the insular cortex and anterior cingulate cortex (Huang et al., 2025). Through these pathways sensory signaling from the has the potential to influence the salience network and limbic circuitry that regulate mood and emotional responses.

The vagus nerve serves as a first line of defense in protecting the airways from injury (Carr and Undem, 2003). In this regard, afferent nerves in the airways detect physical and chemical changes which are then communicated to central networks controlling breathing patterns, efferent output to respiratory and cardiovascular systems, and activation of protective immune responses. Based on their electrophysiological response to stimuli, vagal afferents are classified into two broad categories- myelinated fast-conducting A-fibers and unmyelinated C-fibers (Carr and Undem, 2003).

A-fibers express mechanosensitive rapidly adapting (RAR) and slowly adapting (SAR) receptors (Lee and Yu, 2014). RAR and SAR receptors are sensitive to changes in the mechanical properties of the airways, responding to changes in air flow, lung volume, and pressure. A-fibers activate reflexes that regulate rate and depth of breathing, bronchomotor tone and airway secretion. Thus, stimulation of RAR can induce protective responses including cough, mucous hypersecretion, and bronchoconstriction (Lee and Yu, 2014). Conversely, activation of SARs induces airway dilation and inhibits expiratory muscle activity.

C-fibers comprise the majority of the vagal airway afferents, innervate the entire respiratory tract and project to the nucleus of the solitary tract (Carr and Undem, 2003; Lee and Yu, 2014). C-fibers are considered nociceptive and are readily activated by noxious chemicals, including cigarette smoke and inflammatory mediators. Activation of C-fibers elicits a variety of central and peripheral reflex pathways triggering responses such as airway constriction, mucous secretion, cough, tachypnea, hypotension, bronchial edema, and activation/chemotaxis of inflammatory cells (Carr and Undem, 2003; Taylor-Clark and Undem, 2011; Lee and Yu, 2014).

Central projections of C-fiber and A-fiber neurons appear to innervate distinct NTS subnuclei in a way that is highly regionalized and non-overlapping suggesting they engage different higher order neural circuits linked to physiological function (Kim et al., 2020).

### 3.3 Neuroendocrine cells

Another key component of lung sensory function, that acts in concert with the nervous system, is the airway epithelium and, more specifically, specialized neuroendocrine cells within the epithelium. Pulmonary neuroendocrine cells (PNEC) are a rare epithelial cell type, representing around 0.4% of total lung epithelial cells, that exhibit neuronal and endocrine characteristics and express canonical neuroendocrine markers, such as chromogranin A and synaptophysin (Van Lommel, 2001; Garg et al., 2019). PNEC contain dense core vesicles and have the ability to secrete bioactive neuropeptides, amines, purines and growth factors including gastrin releasing peptide (GRP), serotonin (5-HT), Calcitonin Gene Related Peptide (CGRP), Gamma-aminobutyric acid (GABA) and Adenosine triphosphate (ATP) (Dey and Hoffpauir, 1986; Keith et al., 1991; Adriaensen et al., 2003; Linnoila, 2006; Yabumoto et al., 2008). PNEC can exist as solitary cells or aggregated along with Club-like cells into distinct corpuscles, termed neuroepithelial bodies (NEB). While solitary PNEC are found in the respiratory epithelium of the upper and lower airways, the NEB are confined to the intrapulmonary airways and alveoli but are found most often at airway branch points (Van Lommel, 2001).

PNEC are thought to play an important role as sentinels of the inhaled environment and are known to respond to mechanical stretch, O_2_/CO_2_, and nicotine with the release of neurotransmitters/neuropeptides (Van Lommel, 2001). There are also several members of the olfactory receptor family expressed on PNEC, and challenge of the airway epithelium with volatile compounds or odorant chemicals decreases the 5-HT content of PNEC and increases CGRP release, suggesting a broad chemosensory role for these cells (Gu et al., 2014). The accumulation of PNEC at branch points is suggested to aid in sensing lung volume changes, hypoxia and pathogens or particulates in inhaled air.

PNEC are unusual among airway epithelial cells in being directly contacted by neurons, suggesting they are the airway equivalent of enteroendocrine cells in the gut. PNEC are selectively innervated by myelinated A-fiber type sensory afferent and efferent neurons (Adriaensen et al., 1998; Adriaensen et al., 2006; Brouns et al., 2009; Lembrechts et al., 2011). Sensory nerves innervating PNEC have cell bodies in the vagal ganglion and are characterized by expression of CGRP and purinergic receptors (Adriaensen et al., 1998; Brouns et al., 2003; Adriaensen et al., 2006; Brouns et al., 2009; Lembrechts et al., 2011; Lembrechts et al., 2013). While it has long been assumed that PNEC act as important chemosensory intermediaries between the airway lumen and sensory neuron signaling to the central nervous system (CNS), definitive evidence of this has only been reported recently (Schappe et al., 2024; Seeholzer and Julius, 2024). Utilizing a combination of electrophysiology optigenetics, live cell imaging, anatomical mapping and targeted neuronal in mouse models it was demonstrated that PNEC signaling to sensory neuron that activate centrally mediated protective reflexes is essential to cough-like responses to noxious stimuli (Seeholzer and Julius, 2024) and the gasping response to airway closure (Schappe et al., 2024). However, there is still limited knowledge of how the PNEC response to distinct environmental stimuli initiates vagal signal transduction to the CNS. As the NTS is the predominant target of airway sensory neurons activated by PNEC (Schappe et al., 2024) it will be for future studies to understand how subpopulations of NTS neurons, responsive distinct airway signals, differentially communicate with breathing control circuits.

Another type of chemosensory epithelial cell, distinct from PNEC, are brush cells. Characterized by an apical tuft of microvilli, brush cells, also termed tuft cells, are found as solitary cells throughout the lower airway epithelium (Pan et al., 2020; Billipp et al., 2021; Strine and Wilen, 2022). Brush cells were identified as chemosensory based on their close interaction with nerve endings in the trachea and nose. Brush cells are believed to be involved in monitoring the composition of airway lining fluid, communicating information to sensory nerve endings via cholinergic transmission (Saunders et al., 2013). The chemo-sensing ability of brush cells is principally associated with the expression of taste receptors (Hollenhorst et al., 2022; Hollenhorst et al., 2020), particularly the bitter taste receptor, transient receptor potential melastatin 5 (TRPM5) (Kaske et al., 2007). Brush cell detection of bitter compounds in the airway lining fluid initiates protective respiratory nerve reflexes. Specifically, brush cell–derived acetylcholine and downstream signaling to sensory neurons lead to a reduction of respiratory rate (Krasteva et al., 2011) and increased mucociliary clearance (Hollenhorst et al., 2023).

### 3.4 The sensory immune system

The immune system can also be regarded as a contributor to lung sensory functions; receiving and processing specific information from the environment and initiating effector actions that enable an adequate defensive response (Dozmorov and Dresser, 2010). While initiated locally, these immune responses may not be restricted to the lung.

The concept of a common mucosal immune system is based on the observations that activated lymphocytes migrate from one mucosal site to another (Clancy and Am, 2023). More recently, evidence indicates that immune cells from mucosal sites, such as the airways, can also migrate to influence the CNS. Studies of experimental autoimmune encephalomyelitis (EAE), a rodent model of multiple sclerosis, indicate that the lung can serve as a location where autoreactive T cells become reactivated and gain a migratory phenotype that enables the trafficking into the CNS (Odoardi et al., 2012; Blackmore et al., 2017). Within the lung, chemokine signals direct T cells into bronchus-associated lymphoid tissue (BALT) before they re-enter the blood circulation through mediastinal lymph nodes (Odoardi et al., 2012). Preventing the trafficking of leukocytes through the lung reduces immune cell entry into the brain and delays the onset and reduces the severity of EAE (Park et al., 2024).

As outlined above, there are clear neural, endocrine and immune pathways of communication between the airways and the brain, much as has been described in relation to the gut-brain axis. Sensory signal perception by the brain is a key determinant of behavior and there is growing evidence that aberrant communication between nervous, immune and endocrine systems and the brain leads to mood and behavioral disorders. Below we discuss the influence of sensory signals from the airway on brain and behavior and evidence that pathological changes in the airway can impact mental health.

## 4 The airways and mental health

### 4.1 Olfaction and behavior

The sense of smell is ancient, with general features of the olfactory system shared by all vertebrates (Poncelet and Shimeld, 2020), and provides the ability to perceive and identify critical environmental cues essential for survival. Olfactory dysfunction, which has been linked to mood and neurological disorders such as depression, anxiety, and schizophrenia, demonstrates the profound impact that sensory airway signals can have on mental health. Behaviors such as food seeking, mating, partner selection, threat evasion, detection of fear, and expression of anxiety or aggression are initiated by the detection of odorants and pheromones (Mayhew et al., 2022). Certain responses to aversive or attractive odors are innate and initiate appropriate context dependent behaviors, while there is also adaptation of behavior as a result of experience-based learning and memory of new olfactory associations (Kaur et al., 2014).

The determinants of behavioral responses to specific odors are unclear. However, the olfactory system can be divided, anatomically and molecularly, into subpopulations of OSN and it is suggested that each subpopulation of neurons might be responsible for different behavioral responses to distinct odors (Jiang et al., 2024). Specific behavioral responses have also been attributed to individual chemosensory receptors. TAAR4 elicits avoidance behaviors in mice in response to 2-phenylethylamine, an odorant produced at higher levels in carnivores compared to other animals (Liberles, 2015). Similarly, MS4A1, more commonly known as CD20, a co-receptor for B-cells in lymphocytes, was recently demonstrated to sense predator odorants leading to innate, unlearned avoidance behaviors in mice (Jiang et al., 2024). Conversely, TAAR5 mediates the attraction of mice to trimethylamine, an odorant found in male mouse urine and contributes to within species social interactions (Liberles, 2015). Thus, the olfactory system, via classical OR, or non-classical odorant/chemical receptors, is a major contributor to mammalian behavioral responses to environmental cues.

There is also some evidence for the potential role of olfaction in the perception and expression of feelings and emotions, in empathy-like behaviors and the physiological responses of “emotional contagion.” Evidence for this includes the demonstration that immune changes in tumor-bearing mice are shared with tumor-naïve cage-mates as a result of olfactory signal communication (Alves et al., 2012). In another study, hyperalgesia was transferred from mice subjected to severe persistent inflammation to bystander mice housed separately in the same room (Smith et al., 2016). Crucially, hyperalgesia was also transferred to mice housed in a separate room but given the bedding of the mice with inflammation, suggesting that the transfer of enhanced pain perception was due to odorant signals (Smith et al., 2016). Similarly, although not confirmed, olfactory signals are thought most likely to explain the more recent observation that pups subjected to immune challenge induce inflammatory mediators in both the brain and the periphery of dams (Castany et al., 2024). These immune changes are accompanied by an increase in maternal behaviors and corticosterone levels, and do not require physical interaction between pup and dam (Castany et al., 2024).

Studies also suggest that humans can perceive emotional information from the body odors of other individuals and that exposure to an odor produced during an emotional event can elicit a partial duplication of the associated emotional responses in the perceiver (Semin and Groot, 2013; de Groot et al., 2014; de Groot et al., 2015a; de Groot et al., 2023). For example, detection of body odors produced in response to fear or stress can produce a state of vigilance (de Groot et al., 2015b) while perception of odors produced during positive emotions, such as happiness, can induce facial expressions of happiness (de Groot et al., 2015a). Correspondingly, olfactory impairment has been linked to negative effects on mood and social interactions (Kohli et al., 2016; Rochet et al., 2018). Olfactory deficits have also been identified as indicators of several neurologic diseases, including multiple sclerosis, epilepsy, Alzheimer’s and Parkinson’s disease (Barresi et al., 2012; Rahayel et al., 2012) and impaired identification of odors is a characteristic of patients with schizophrenia, the early onset of psychosis and individuals at high risk of psychosis (Lin et al., 2015).

In addition to olfaction, there is also evidence that trigeminal nerve signaling influences mood and behavior. Trigeminal neuralgia has been linked with increased risk of developing major depressive disorder, anxiety disorder, and sleep disorder, while trigeminal nerve stimulation has been applied clinically to a variety of psychological conditions including MDD, PTSD and ADHD (Generoso et al., 2019; Shiozawa et al., 2014; Cook et al., 2016).

### 4.2 Mood disorders and lung disease

Much has been made of the impact of the gut-brain axis on mental health and the bi-directional relationships that link mood with functional and inflammatory bowel disorders. However, there is also good epidemiological support for a lung-brain influence on mental health, with an association between lung diseases, such as asthma and COPD, and disorders including anxiety, depression and posttraumatic stress disorder (PTSD).

Asthma is characterized by chronic airway inflammation and hyperresponsiveness, mucus overproduction and airway obstruction (Barrios and Ai, 2018). Comorbidity between asthma and mood disorders is a consistent observation and there is evidence the relationship goes beyond the psychosocial impact of living with a chronic health condition (Jiang et al., 2014; Bedolla-Barajas et al., 2021; Alanazi et al., 2022). This bidirectional relationship may reflect shared immune-inflammatory pathways, with airway inflammation potentially driving neuroinflammation and mood disturbances, while psychological stress can exacerbate airway hyperresponsiveness and inflammation (Wang et al., 2023). The relationship between asthma and mood disorders also appears to exist throughout life, from childhood to old age. Adolescents with asthma are twice as likely to have anxiety and/or depression compared to peers without asthma (Chen et al., 2014; Dudeney et al., 2017). In the elderly, asthma has been associated with a higher prevalence of depression even when adjusting for psychosocial factors and physical comorbidities, and controlling for other chronic illnesses (Ng et al., 2007). Reciprocally, anxiety and depression are both strong predictors of poor asthma control and are associated with more emergency department visits. Asthma hospitalization rates and subsequent length of hospital stay are also increased in individuals with comorbid anxiety and/or depression when compared to those without these concurrent conditions (Kullowatz et al., 2007; Munoz et al., 2020; Kulikova et al., 2021). This close correlation between mood disorders and poor asthma outcomes suggests that the relationship between asthma and mood disorders is bi-directional.

Beyond depression and anxiety, there have been multiple reports of an association between asthma and PTSD. PTSD is characterized by negative mood, intrusive memories, avoidance behaviors, and altered arousal behaviors such as increased irritability usually associated with prior exposure to a traumatic event (Lancaster et al., 2016). While inflammation in general has been linked to PTSD, there is evidence of a particularly strong link between asthma and this mental health disorder (Goodwin et al., 2007; Xu et al., 2016; Wisnivesky et al., 2021). A study of war veterans identified that those with the highest PTSD scores were twice as likely to have asthma than those without PTSD, independent of demographic, familial or genetic factors (Goodwin et al., 2007). Increased prevalence of asthma has also been reported in those who developed PTSD following the 9/11 terrorist attack (Shiratori and Samuelson, 2012) and in a large longitudinal population study (Hung et al., 2019). PTSD is one of the greatest risk factors for a decrease in asthma related quality-of-life (Chung et al., 2012; Wisnivesky et al., 2021), most likely due to poorer asthma self-management behaviors (Chung et al., 2012). Individuals with asthma that develop PTSD have been demonstrated to experience a subsequent aggravation of asthma symptoms, while non-asthmatic subjects who develop PTSD have increased risk of adult-onset asthma, again suggesting a bidirectional relationship between the lung disease and mental health (Wisnivesky et al., 2021).

While asthma may be the most studied lung disease with regard to relationships with mood disorders, COPD, another major chronic inflammatory disease of the airways, is also associated with changes in brain function, mood and behavior. COPD is a heterogeneous group of progressive, inflammatory lung conditions, including chronic bronchitis and emphysema, characterized by persistent respiratory symptoms and airflow limitation (Global Initiative for Chronic Obstructive Lung Disease, 2019). Up to half of the COPD patient population regularly experience symptoms of depression (van den Bemt et al., 2009; Schneider et al., 2010; Volpato et al., 2021). As with asthma, occurrence of depression in COPD patients is associated with poorer health-related quality of life and an increased risk of hospitalization and length of hospital stay. COPD with co-morbid depression is also associated with an increase in mortality compared to patients without depression (Mullerova et al., 2014; Volpato et al., 2021; Smith et al., 2025). Again, as with asthma, evidence indicates that the relationship between depression, and COPD is bidirectional with depression negatively influencing prognosis in COPD while COPD increases the risk of developing depression and/or anxiety.

While the observed bi-directional associations between lung disease and mood disorders is indicative of a lung-brain axis, the relationship between these comorbidities is complex. Confounding factors such as psychosocial factors of living with a chronic disorder and potential impact of common treatments, such as corticosteroids, cannot be discounted (Ritz et al., 2025). However, in addition to epidemiological relationships, human neuroimaging studies and animal models point to causal biological mechanisms linking inflammatory lung disease and changes in brain structure and function.

### 4.3 Airway disease and brain structure

In addition to altered mood and cognition, inflammatory lung diseases are associated with anatomical changes in the brain. The hippocampus plays an important role in cognition and in the pathophysiology of mood disorders, with hippocampal atrophy a characteristic of chronic depression (Tai et al., 2021). In a large sample of middle-aged subjects with asthma, hippocampal volumes were found to be reduced compared to matched controls (Carlson et al., 2017). In another study, hippocampal volumes of young adults with asthma were not altered compared to controls but hippocampal neural integrity was lower and certain aspects of the disease were associated with changes in basal ganglia structures linked to processing anxiety and fear (Ritz et al., 2020). Specifically, lower volumes of the putamen and pallidum were associated with longer asthma duration and poorer disease control (Ritz et al., 2020). Longer asthma duration has also been associated with increased volume of the periaqueductal gray (von Leupoldt et al., 2011) a brain region involved in antinociception (Irvine et al., 2024) and behaviors associated with fear and defensive actions (Melleu and Canteras, 2025), while a recent study demonstrated structural brain changes in participants with asthma in multiple regions associated with reward and salience networks (Carrol et al., 2025). COPD has also been associated with altered gray matter volumes of cortical and subcortical structures (Zhang et al., 2012; Esser et al., 2016) with structural changes related to disease duration and an association with patients fear of dyspnea and exercise (Esser et al., 2016). The driver of structural brain changes associated with asthma and COPD are unclear but likely reflect the impact of multiple overlapping mechanisms. Both asthma and COPD can cause chronic or intermittent cerebral hypoxia, which is known to affect hippocampal volume, white matter integrity, and global cortical atrophy (Cui et al., 2024). However, in asthma, hypoxia is largely associated with severe disease (Kroll and Ritz, 2023) while brain structure changes are observed even in milder forms (Carrol et al., 2025). Furthermore, while hypoxia is a common complication of COPD, at least one study found no correlation between degree of brain structural changes and either partial pressure of oxygen (PaO2) or oxygen saturation (SaO2) (Yin et al., 2019). Such findings suggest hypoxia may not be the major driver of brain structural changes associated with lung disease.

Peripheral immune signals associated with airway inflammation, including cytokines and lymphocytes, can also influence the brain via circumventricular organs, crossing the blood brain barrier, the permeability of which can be increased in inflammatory conditions, or active uptake. In this way peripheral immune signals have been demonstrated to induce neuroinflammation in certain brain regions (Sun et al., 2022). Neuroinflammation in turn can lead to reduced gray matter volume, decreased dendritic branching, and white matter abnormalities (Chaudhary et al., 2025). Peripheral inflammation predominantly impacts limbic system structures and cortical areas, including the amygdala, hippocampus, prefrontal cortex, hypothalamus, striatum, and insula, (Tan et al., 2023) suggesting circulating immune signals derived from the lungs may contribute to brain changes associated with asthma and COPD. It is also possible that changes in cytokines, metabolites, or microbial signals in the lower airways, which are associated with lung disease, could signal via sensory nerves to the NTS and via projections influence components of the salience network and limbic circuitry.

### 4.4 Mechanistic insights to the link between lung disease and the brain

While studies have identified anatomical and behavioral changes in asthma and COPD, the mechanisms underlying these relationships are unclear. However, there are several studies that provide some insight into lung-brain connections in relation to airway inflammation.

Rodent models of asthma have demonstrated that allergic airway inflammation is associated with anxiety and depressive-like behaviors in addition to cognitive defects such as memory impairment (Caulfield et al., 2017; Tel et al., 2021; Bejeshk et al., 2023). There is strong evidence from these models that airway inflammation drives an inflammatory response in the brain. Multiple studies have identified that anxiety and depressive-like behaviors in models of asthma are associated with increased levels of inflammatory cytokines in the CNS (Antunes et al., 2022; Kanaya et al., 2022). Indicators of microglia, astrocyte and mast cell activation (Kanaya et al., 2022) have also been reported together with increased neural activity in brain regions including the prefrontal cortex, hippocampus, amygdala and hypothalamus (Dehdar et al., 2019; Antunes et al., 2022; Gholami-Mahtaj et al., 2022; Dehdar et al., 2023a; Dehdar et al., 2023b). These findings suggest a mechanistic link between airway inflammation and brain function, potentially mediated by cytokine signaling and microglial activation, which could contribute to mood and cognitive disturbances.

Emphasizing the bidirectional nature of lung-brain signaling associated with airway pathology, the influence of mood and emotion on the airways has been explored in laboratory-based studies of human subjects. Exposure of asthma patients to negative affective stimuli results in clinically relevant bronchoconstriction in a significant proportion of subjects (Plourde et al., 2017; Ritz et al., 2019) and this bronchoconstriction is associated with stronger activation of cingulate cortex areas, in particular the anterior (ACC) and mid (MCC) cingulate cortex regions (Ritz et al., 2019). The airway response to emotional stimuli in individuals with asthma can be eliminated by cholinergic blockade and is independent of airway inflammation or hyperreactivity to methacholine (Ritz et al., 2010), suggesting the mechanism of affective influence on the airways, is CNS-mediated vagal excitation. The level of activity in the ACC and insula cortex to asthma-relevant emotional stimuli was also demonstrated to be positively correlated with markers of inflammation (sputum eosinophil number and TNF levels) and airway obstruction in response to subsequent allergen exposure in asthmatic subjects (Rosenkranz et al., 2016).

Overall, evidence strongly suggests that signaling along the airways-brain axis can impact the pathophysiology of both respiratory diseases and mood disorders. Also, as identified above, altered airway microbiota composition is associated with airway disease. Next, we discuss evidence that microbes engage airway to brain signaling pathways and thus have the potential to alter brain function and behavior.

## 5 Airway microbiota and the brain

### 5.1 Microbes and olfactory development

Exposure to microbes is essential for normal olfactory system development. The olfactory epithelium of germ-free mice is structurally distinct from conventionally housed animals, with a thinner olfactory cilia layer and decreased cellular turn-over (Francois et al., 2016). Functionally, the absence of microbes is associated with an overall increase in the amplitude of responses to odorants and a decrease in transcription of olfactory transduction factors (Francois et al., 2016). There is also evidence that the impact of bacteria in the olfactory system is organism specific. For example, certain microbes, including *Staphylococcus aureus*, but not others, drive differentiation of OSN (Casadei et al., 2019). This microbe driven differentiation involves induction of REST (RE1 silencing transcription factor), a transcription factor that binds to the conserved motif repressor element 1 (RE1) and regulates expression of many neuronal genes (Casadei et al., 2019). These findings suggest that microbial interactions with the olfactory system could have downstream effects on brain function and behavior, making the nasal microbiota an intriguing target for further research.

In keeping with organism-specific effects on OSN development, there is also good evidence that composition of the microbiota influences olfactory function. Different olfactory preferences develop in germ-free mice colonized with distinct microbiota profiles (Naudon et al., 2020). Olfactory epithelium properties, including electrophysiological responses and expression of genes associated with the olfactory transduction cascade also show several microbiota profile specific differences (Naudon et al., 2020). In humans, a study by Koskinen et al. (2018) indicated that subjects with lower olfactory function had a more diverse nasal microbiome compared to those with normal function. Evidence also suggested butyrate-producing *Faecalibacterium* and *Porphyromonas* species may be involved in reduced odor perception and discrimination (Koskinen et al., 2018).

The observations that microbes influence development of the olfactory system suggest that there may be a window of time in early life when microbial exposure directs olfactory development and thus subsequent behavioral response to olfactory signals. However, this research is still in its infancy and the role of distinct nasal microbiota profiles on long-term olfactory responses is currently unknown.

### 5.2 Microbial metabolites and nasal chemosensory systems

In addition to modulating OSN development, it is possible that nasal microbes can influence olfaction, and consequently behavior, through the direct production of odorants. Microbes can generate a number of odorants that bind to classical OR and TAARs (Bienenstock et al., 2018). Microbial metabolites may also influence mood and cognition by altering local olfactory pathways or activating central nervous system circuits via signaling through the olfactory bulb or trigeminal nerve. For example, the volatile amine, β-phenylethylamine (PEA), is synthesized by a wide range of bacteria (Kim et al., 2012; Irsfeld et al., 2013). PEA is an agonist of TAAR1 and TAAR4 (Xie and Miller, 2008; Borowsky et al., 2001) and has been shown to activate components of the hypothalamic – pituitary – adrenal (HPA) axis (Kosa et al., 2000) and drive predator avoidance behavior in mice (Dewan et al., 2013). Another odorant, trimethylamine (TMA), that is extremely attractive to mice but repels rats, is also synthesized by commensal microbes (Liberles, 2009, 2015). Murine attraction to TMA is abolished in TAAR5-knockout mice or by enzymatic degradation by the liver enzyme, flavin-containing monooxygenase 3 (Li et al., 2013). Furthermore, preventing gut microbe-driven choline conversion to TMA in mice alters their olfactory perception and social behavior (Massey et al., 2023). However, while microbial production of behavior modulating odorants has been shown in a variety of mammals, bats and insects (Ezenwa and Williams, 2014), the focus has been on synthesis by intestinal microbes or the microbiota of specialized scent glands, whereas the potential role of nasal microbes in chemo-olfactory signaling is remarkably underexplored.

In addition to producing volatile compounds that engage olfactory signaling, microbes can produce an array of neuroactive compounds including classical neurotransmitters such as serotonin, dopamine and GABA (Lyte, 2013). Furthermore, microbes can also induce neurotransmitter release from host cells (Forsythe and Kunze, 2013; Danhof et al., 2023). Microbe produced or induced neurotransmitters in the sinonasal mucus and epithelium have the potential to signal the CNS via local nerve fibers which include both OSN and trigeminal sensory neurons. The trigeminal nerve, also known as the 5th (V) cranial nerve, has a branch (Maxillary nerve branch) that innervates the nasal cavity and serves as another neural signal pathway to the brain (Shusterman, 2023). Trigeminal chemosensory fibers from the nasal cavity project to the lateral parabrachial complex where signals are then transmitted to the amygdala (Aucoin et al., 2023; Shusterman, 2023). There is evidence that trigeminal nerve signaling influences mood and behavior. Trigeminal neuralgia has been linked with increased risk of developing major depressive disorder, anxiety disorder, and sleep disorder, while trigeminal nerve stimulation has been applied clinically to a variety of psychological conditions including MDD, PTSD and ADHD (Generoso et al., 2019; Shiozawa et al., 2014; Cook et al., 2016).

### 5.3 Microbes and nasal immune system

Changes in the nasal microbiota may also influence the CNS, and consequently mood and behavior, through modulation of the immune system. Anxiety, depression and PTSD have been associated with a proinflammatory state and there is evidence that cytokines and reactive oxygen species, generated during chronic inflammation, can alter neurochemical pathways to impair brain function and affect emotional and cognitive processes. The major chronic inflammatory conditions of the nasal cavity include Allergic Rhinitis (AR) and Chronic Rhinosinusitis (CRS), both of which have been associated with an increased prevalence and incidence of anxiety and depression (Erskine et al., 2017; Rodrigues et al., 2021). There is also evidence of a bi-directional relationship between the nasal microbiome and chronic nasal inflammation.

While some specific details of compositional changes may differ between studies, overall data supports an altered nasal microbiome in AR. The nasal mucosa of adults and children with AR have been demonstrated to have an increase in the *Firmicutes* phylum, specifically the genus *Staphylococcus*, compared to healthy subjects (Gan et al., 2021; Kim et al., 2022; Teng et al., 2024). Furthermore, at least one study has demonstrated that within the genus *Staphylococcus, S. aureus* was most abundant in subjects with AR while *S. epidermidis* was predominant in healthy controls (Kim et al., 2022). This may be clinically relevant as *S. aureus*-secreted toxins can be recognized as superantigens, promoting Th2-mediated inflammation and *S. aureus* is known to elicits the production of inflammatory cytokines including IL-4, IL-5, and IL-13 (Chegini et al., 2022). In contrast, *S. epidermidis* may reduce allergic inflammation by suppressing IL-33 production in nasal epithelium (Jeon et al., 2023). An increase in the genus *Staphylococcus* has also been associated with CRS, particularly CRS with nasal polyps (CRSwNP) (Gomez-Garcia et al., 2024) and *S. aureus* has been hypothesized to play a role in this condition (Shaghayegh et al., 2023). The ability to predict recurrence of CRSwNP based on nasal microbiome composition has been reported (Zhao et al., 2022), while nasal microbiome transplants obtained from healthy individuals and administered as nasal lavages to patients with CRSwNP resulted in significant and lasting reduction of symptoms (Martensson et al., 2023). Among subjects with chronic nasal inflammation there is also evidence that microbiome changes are related to olfactory loss. Subjects with CRS associated with olfactory dysfunction were demonstrated to have decreased diversity in the nasal microbiome compared to those with CRS and normal olfactory function (Han et al., 2023). These changes in microbiota diversity may impact odor perception and downstream signaling to the brain, potentially influencing emotional processing and quality of life. The diversity differences were associated with significant enrichment of *Acinetobacter johnsonii* in those with olfactory loss while abundance of *Mycoplasma arginini*, *Aeromonas dhakensis*, and *Salmonella enterica* was significantly reduced (Han et al., 2023). Metagenomic analysis suggested the changes in microbiome composition were associated with increased purine metabolism (Han et al., 2023). Overall, studies support links between nasal microbiota changes, upper airway inflammation and olfactory disruption. Furthermore, both inflammation and impaired olfaction have been associated with mood disorders. However, a direct line of causality between inflammation associated changes in nasal microbiota and brain function has yet to be established.

### 5.4 Nasal microbial endocrinology

The intriguing possibility that nasal microbes may modulate levels of hormones in the nasal cavity that in turn influence behavior was uncovered in a recent study (Xiang et al., 2025). Nasal microbiome analyses of human subjects identified a positive correlation between depression scores and *S. aureus* abundance. A causal relationship was then suggested by the observation that nasal microbiota transplants from depressed subjects reproduced depression-like behavior in mice and maintained the differential abundance of *S. aureus.* Further investigation revealed sex hormone-degrading enzyme, 17β-hydroxysteroid dehydrogenase, expressed in *S. aureus* that degraded testosterone and estradiol in mice, and this activity was associated with lower levels of dopamine and serotonin in the murine brain. Nasal inoculation with 17β-hydroxysteroid dehydrogenase deleted *S. aureus*, failed to induce depressive like behavior or the reduction in dopamine and serotonin (Xiang et al., 2025). This is the strongest evidence, to date, of a direct impact of nasal microbes on depressive-like behavior, but clearly such studies need to be replicated. Microbial endocrinology; the ability of microbes to modulate host hormone levels and signaling, is a growing field (Li et al., 2022; Cotton et al., 2023; McCurry et al., 2024) and may prove a fruitful avenue of investigation in relation to the airway-brain axis.

### 5.5 Lung microbiota and the brain

While very few studies have investigated the implications of changes in lung microbiota composition for the CNS, there is supporting evidence for a microbiota-lung-brain axis. In a murine EAE model, distinct shifts in the lung microbiome composition had divergent influence on the susceptibility of rats to develop autoimmune disease of the CNS (Hosang et al., 2022). Treatment of the lungs with neomycin resulted in a shift in the microbiota that increased the relative abundance of lipopolysaccharide-enriched phyla and was associated with a type-I-interferon-primed state in microglial cells resident in the brain (Hosang et al., 2022). The response of these microglia to autoimmune-directing type II interferons was impaired, which led to a decreased immune cell recruitment to the brain and reduced clinical signs of EAE. Conversely, suppressing lipopolysaccharide-producing lung phyla with polymyxin B led to disease aggravation. These findings strongly suggest that the lung microbiome can regulate brain autoimmunity and may be involved in brain autoimmune diseases (Hosang et al., 2022). This bidirectional relationship highlights the potential for lung microbiota changes to influence not only neuroinflammation but also mood and behavioral disorders, further emphasizing the lung as a critical site for brain-body interactions. The cellular and molecular mechanisms underlying the impact of the lung microbiome on brain autoimmunity, and whether this is related to the role of the lung as a niche for autoreactive T cells, remains to be determined (Odoardi et al., 2012; Blackmore et al., 2017). These observations may also have implications beyond autoimmune disease. Given that neuroinflammation and microglia activation have been associated with anxiety, depression and PTSD (Sild et al., 2017; Sah and Singewald, 2025), the observation that the lung microbiota composition can influence brain immune function provides a possible mechanism through which these organisms influence the susceptibility and severity of mood disorders.

Neuroendocrine cells in the lung are also known to act as chemosensors for bacteria derived signals. PNEC express multiple OR (Gu et al., 2014), indicating that these cells can respond to the same volatile compounds and odorants as OSN, and have the ability to signal to the brain directly via synapses with vagal sensory neurons (Garg et al., 2019). Mouse and human PNEC have been shown to release the immunomodulatory neuropeptides CGRP and substance P in response to the common aeroallergen, house dust mite (HDM) (Sui et al., 2018; Mann-Nuttel et al., 2025). The human PNEC response to HDM is dependent on protease activated receptor (PAR)-1 suggesting protease detection is part of the sensory repertoire of these cells (Mann-Nuttel et al., 2025). This may be significant to host-microbe signaling as many bacteria, including *S. aureus* utilize PAR-1 activating proteases as mediators of virulence and pathogenicity (Kline et al., 2024). In the skin *S. aureus* derived proteases activate PAR-1 on sensory neurons to induce an itch response (Deng et al., 2023). While not demonstrated to date, it seems likely that PAR-1 expression on PNEC form part of the lungs microbe surveillance system with a direct neural pathway to the brain.

The immune system is also involved in the response to neuroendocrine sensory signaling in the lung. In mice, a mutation in the Roundabout (Robo) receptors, Robo1/2, results in increased neuropeptide secretion from PNEC and an associated increase in airway neutrophils, eosinophils, macrophages, and T cells (Branchfield et al., 2016). This airway inflammatory response in Robo1/2 mutant mice is prevented by a PNEC specific knock-out of the neuropeptide calcitonin gene related peptide (CGRP) (Branchfield et al., 2016). Conversely, mice deficient in PNEC are resistant to developing allergic airway inflammation following allergen challenge (Sui et al., 2018). In this model, the CGRP released from PNEC following allergen challenge in mice activates ILC2 to drive type 2 immune responses (Sui et al., 2018).

Tuft cells, express the bitter taste receptor, TRPM5, that can be activated by several bacterial quorum-sensing molecules (QSM) (Hollenhorst et al., 2020; Hollenhorst et al., 2022). QSM are signal molecules released and perceived by bacteria to coordinate cooperative behaviors such as increasing population size and virulence (Fuqua and Greenberg, 1998). Brush cell recognition of QSM from *P. aeruginosa* results in a substance P– and sensory neuron dependent increase in lung vascular permeability and neutrophil influx (Hollenhorst et al., 2022). This response suggests that brush cells augment the ability of sensory neurons to detect the presence of bacteria. As bacteria such as *P*. *aeruginosa* use QSM to communicate and increase numbers and virulence, the brush cell recognition system may, together with PNEC, be an “early warning” sensor of bacterial invasion, instigating protective responses before bacteria reach critical numbers for infection.

As was once the case for the gut, our knowledge of airway-microbe interaction relates almost exclusively to pathogens and disease-causing organisms. How commensals engage with the host adaptive systems in the airways is largely unknown but likely has many parallels with the gut, where microbes induce mechanisms of active tolerance in the host to maintain a mutually beneficial relationship (Sansonetti, 2011). Considering bi-directional aspects of immune-microbe interactions and particularly suggestions from GWAS the immune-related polymorphisms shape airway microbiota (Igartua et al., 2017; Losol et al., 2021) it can be speculated that polymorphisms that shift airway community structure could produce persistent microbe mediated peripheral signals that bias neurodevelopmental trajectories or adult brain function. Such microbe derived signals could include, *inter alia*, volatile compound that change olfactory cues and activate vagal/trigeminal circuits and induced cytokine responses leading to chronic low-grade inflammation that has been associated with mood disorders (Teixeira et al., 2025). There are multiple genes involved in are immune/barrier function that are plausible candidates for shaping airway microbiota with downstream neuroimmune impact. These genes include, but are certainly not limited to, Toll-like and NOD-like receptors, which are involved in microbial pattern recognition and influence host immune responses; MUC5AC and MUC5B, which encode major airway mucins—proteins that affect mucus viscosity and clearance, thereby impacting microbial colonization; and β-defensins, antimicrobial peptides that help shape the structure of microbial communities on mucosal surfaces (Meade and O’Farrelly, 2018).

Future research should aim to deepen our understanding of host influences on airway microbiota composition as well as the specific contributions of airway-resident commensals to brain health and behavior. This includes identifying key microbial species and pathways that could serve as targets for therapeutic intervention.

### 5.6 Therapeutic potential of the microbiota-airway-brain axis

Probiotics have been a major focus of strategies aimed at exploiting the therapeutic potential of the gut-brain axis. This is an attractive approach as non-pathogenic, generally regarded as safe (GRAS) organisms, with potentially beneficial immune and neuromodulatory activity have been identified and are easily administered to the target site orally. Targeting the lower airway microbiome with probiotics involves additional challenges related to safety of inhaled preparations, with the risks of infection or excessive immune response poorly understood. However, there have been clinical trials of intranasal probiotic sprays or drops (Martensson et al., 2016; Martensson et al., 2022; Folino et al., 2024). While these treatments had limited efficacy against nasal inflammation the studies did demonstrate probiotic preparations can be administered safely, Direct airway delivery of probiotics has also been investigated in several preclinical studies (Glieca et al., 2024). To date, there has been a focus on *Lactobacillus and Bifidobacteria* strains as probiotics, with the assumption these organisms will maintain, or even have enhanced, benefits locally compared to those observed with oral delivery. This is supported by at least one study demonstrating that intranasal administration of *L. paracasei* was more efficient than intragastric administration in decreasing allergic airway inflammation in mice (Pellaton et al., 2012). However, better knowledge of microbe-host interactions in the airways may lead to the identification of bacteria beyond the *Lactobacillus and Bifidobacteria* genera that could provide health benefits when administered through inhalation. When it comes to targeting the lower airways, the potential for microbes in the oral cavity to seed the lung microbiota (Proctor and Relman, 2017) suggests, that alternatives to inhalation such as probiotic chewing gum could be explored. Additionally, the safety and technical challenges of inhaling whole viable bacteria could be avoided through the use of prebiotic preparations that provide nutrients or otherwise establish a favorable environment for beneficial organisms and/or suppress growth of detrimental microbes. Identifying microbial metabolites that modulate chemosensory signaling between the airways and brain could also lead to development of what have been termed “postbiotic” treatments. Here again a greater understanding of both the microbial signals and corresponding sensors in the airways is needed. While there are major gaps in our knowledge and technical hurdles to overcome, the exploration of microbial therapies tailored for the airways, including probiotics, prebiotics, and postbiotics, could revolutionize treatments for respiratory diseases and mental health conditions alike.

## 6 Discussion

Multiple airway-brain communication pathways exist and there is evidence that these pathways can be engaged by microbes in the nasal cavity (Figure 2) and lungs (Figure 3).

From an evolutionary point of view there is a clear rationale for the existence of an airway-brain axis. The nose and upper airway are a primary route for inhaled pathogens, toxins and environmental cues. Rapid detection and an ability to direct information to motor/behavioral centers and to immune effectors confers selective advantage. In particular, olfactory nerve pathways give a relatively direct neuronal route from environment to the CNS enabling immediate behavioral responses. In insects, olfactory signals, largely volatile organic compounds, from microbes in the environment can influence foraging, egg laying, and aversive/threat avoidance behaviors (Moyano et al., 2023; Zhang H. et al., 2024). Symbiotic microbes can also alter olfactory sensitivity in insects modifying behavior to benefit microbial propagation and survival. It is not difficult to envision that such signaling mechanisms have developed to help maintain microbe-host symbiotic relationships in other animals including mammals.

While a rationale for microbes in lower airways directly influencing affective behaviors may seem less obvious, microbes are key indicators of environmental conditions and, again, hosts that distinguish harmless commensals from harmful invaders have survival advantages. Neuroendocrine cells in fish gills have recently been demonstrated to act as chemosensory links between the environment and immune defense (Hussein et al., 2025) and there is increasing evidence that mammalian lung neuroendocrine cells play the same role (Sui et al., 2018; Mann-Nuttel et al., 2025). Furthermore, in mammalian lungs, chemosensory cues from commensal microbes, acting via neuroendocrine cells, likely contribute to interoceptive signaling systems allows the brain to gauge the physiological state of the lower airways. Disruption of interoceptive signaling, or failure of centrally coordinated responses to correct suboptimal conditions indicated by interoceptive signals, has been suggested to contribute to anxiety and depression (Paulus and Stein, 2010; Verdonk et al., 2025). In this regard commensal bacteria could provide tonic signals (via microbial metabolites and low-level microbe associated pattern recognition receptors stimulation) that indicate airway “safety” calibrating behavior appropriately and maintaining host–microbe mutualism. Such a relationship would benefit both the host (defense, energy economy, social fitness) and the microbes (niche preservation, transmission).

While this review focused on the nasal cavity and lungs, it should be noted sensory communication with the brain occurs throughout the airways. For example, it has been demonstrated that prostaglandin E2 responsive sensory projections from the glossopharyngeal (ninth cranial) nerve to the nasopharynx mediate sickness behavior in response to influenza-induced sickness behavior in mice (Bin et al., 2023). However, there are several knowledge gaps that must be addressed before the respiratory system is positioned alongside the gut as a crucial host-microbe interface with a meaningful influence on brain function, mood, and behavior. While associations have been observed between altered airway microbiota and respiratory diseases like asthma and COPD and between airway inflammation and mental health disorders such as anxiety, depression, and PTSD, establishing causal relationships remains a key area for future research. Understanding the baseline composition of a healthy airway microbiome, and corresponding metabolome, is essential to distinguishing meaningful disruptions linked to disease and mental health conditions. In parallel, more studies of microbiota composition in the airway of subjects with mood disorders, or those with respiratory disease and co-morbid mental health conditions, are needed. Better understanding of the airway microbiome composition in these conditions may allow for the development of microbe-based biomarkers, either for mood or cognitive disorders alone, or to identify those subjects with inflammatory lung disease at most at risk for CNS co-morbidities. However, such studies are hampered by a lack of standardized guidelines and research methodologies for the collection of airway microbiome samples. Sample collection ranges from the use of swabs and nasal rinses, for the upper airways, to bronchoalveolar lavage fluid (BALF) and induced sputum collection for the lower airways. Such variables, combined with a further lack of standardization in analysis pipelines, result in difficulties comparing studies and reproducing findings that could identify signature profiles or key constituents of the airway microbiome influencing mental health. The low microbial biomass in the lungs presents specific challenges for sequencing-based studies. The low number of resident microbes makes these samples highly susceptible to contamination, from reagents, equipment, or the upper airways during sample collection, which can mask the true microbial composition. Consequently, careful methodological controls, such as rigorous decontamination protocols, analytical approaches that identify likely contaminant sequences, inclusion of extraction and environmental negative controls and, crucially, independent reproduction of study findings are essential to allow for reliable assessments of the lung microbiome. Establishing standardized guidelines for sample collection and analysis is crucial to enable robust comparisons across studies and advance our understanding of the role of the airway microbiome in mental health.

This review largely focused on the interactions of host and bacteria in the airways, but it is important to note that both commensal viruses and fungal organisms are as likely to influence airway brain communication and studies of the virome and mycobiome are needed.

Priorities for future investigations to confirm the existence of and microbiota-airway-brain axis (Figures 2, 3) include the use of animal models to demonstrate a clear link between targeted disruption of the airway microbiome and changes in behavior. Targeted use of antibiotics, probiotics or microbial transplants in mammalian models with subsequent assessment of brain chemistry and behavior, as recently described by Xiang et al. (2025) will be good first steps in this process. Similarly, assessment of shifts composition of nasal and lung microbiome in response to psychological stressors in animal models would help confirm the bidirectional nature airway-brain signaling. In this regard, new insights into airway microbe interactions with efferent signaling from parasympathetic and sympathetic nerves (De Virgiliis and Di Giovanni, 2020) will likely be key to understanding any CNS driven changes in lung microbiome composition.

More preclinical studies, such as those of Hosang et al. (2022), establishing proof of principle for mechanistic links between respiratory microbes and brain function are also needed. Overall, our knowledge of the sensory systems of the airways and their interactions with luminal content, including microbes, lags that of the gut. However, innovative approaches including lung organoids and epithelial cell cultures enriched for chemosensory cells (Candeli and Dayton, 2024; Mann-Nuttel et al., 2025), potentially in 3D co-culture systems with neurons (Malheiro et al., 2021; Goldsteen et al., 2022), will allow for hitherto unobtainable mechanistic insights to host-microbe interactions in human airways. Similarly, animal models utilizing combinations of optogenetics and Designer Receptors Exclusively Activated by Designer Drugs (DREADD)-based chemogenetic tools may allow for dissection of pathways linking microbes, airway sensory nerves and brain circuitry involved in behavior (Roth, 2016; Seeholzer and Julius, 2024). Dual approaches of human microbiome analysis and mechanistic studies will also be required to identify airway microbes that may have beneficial or detrimental effects on brain and behavior, knowledge that may in turn be used to develop therapeutic strategies.

In conclusion, the nasal cavity and lungs, beyond their primary role in respiration, function as sensory organs with intricate communication pathways to the brain. How the diverse microbiota of the airways influence lung-brain communication is currently poorly understood. A combination of state-of-the-art preclinical models and standardized human microbiome studies are required to advance this field. A better fundamental understanding of host-commensal interactions in the airways would allow for the assignment of viable mechanisms of action that may contribute to establishing causal relationships between observed microbiota changes and altered airway-brain interactions. Such insights could place the airways alongside the gut as a key facilitator of microbial communication with the brain. Future research, aimed at unlocking the full therapeutic potential of the microbiota-airway-brain axis, could transform how we approach the prevention and treatment of complex diseases at the intersection of respiratory and mental health.
